# Editorial: Investigating the human brain and muscle coupling during whole-body challenging exercise

**DOI:** 10.3389/fphys.2015.00285

**Published:** 2015-10-20

**Authors:** Stephane Perrey

**Affiliations:** EuroMov, University of MontpellierMontpellier, France

**Keywords:** fatigue, neuroimaging, pain, sensation, peripheral feedback, perception of effort

Research into correlates and determinants (causal relationship) of fatigue has burgeoned in the past two decades. However the link between cortical activity and muscle force generation on fatigue that develops during exercise is not very well understood. While there is extensive evidence suggesting that the central nervous system can contribute to the inability of a human being to sustain a motor task, some questions are unsolved. Is fatigue a sensed variable? Could substantial progress in neurophysiological techniques have additional benefits in the understanding of fatigue? What have we learned and what happens next? Answers require likely the combined insights not only from physiologists, but of neuroscientists and psychologists. Recent research in this area presents three trends providing current interests and opinions in the field.

## The perception of fatigue

The first issue concerns the fatigue state with respect to changes in motor performance. The picture of fatigue that emerges is the conscious perception of a sensation of fatigue according to the phenotypic traits of the individuals mediated by environment and culture (Boullosa and Nakamura, 2013). In this respect, muscle fatigue modifies the ratings of perceived exertion (RPE) required to maintain task performance and reduces exercise tolerance independently from metabolic stress (Marcora et al., 2008). During the fatigue state, brain is able to sense exercise intensity and environmental conditions. In this context, exercise-induced pain is proposed as a likely candidate contributing to the sensation of fatigue in exercise performance (Mauger, 2013). Athletes' attitudes toward pain and the cognitive strategies they use while experiencing pain, may be reflected in their pain tolerance levels and their performance.

The role of a second sensation brought by exercise with respect to pain is the sense of effort. The latter is centrally generated and accompanies peripheral sensory feedback on self-selected exercise intensity. While, subjective awareness of effort required to perform an exercise task can be dissociated from the physical discomfort induced by various types of exercise (e.g., sub-maximal constant-effort cycling and brief sub-maximal and maximal cycling bouts in Christian et al., 2014). Here, at the core of many integrative models explaining fatigue with the central nervous system highly involved in exercise performance regulation (Boullosa and Nakamura, 2013) is the concept of fatigue as a sensation or emotion (Noakes, 2012).

## The exercising brain

Probably, development of fatigue state is dependent on the short-term reorganization of the prefrontal—motor network during exercise. Methods to challenge this idea are now emerging. Imaging the brain during increased physiological demand (i.e., exercise) with sufficient ecological validity (Mauger, 2013) is a rapidly growing field.

Advances in functional neuroimaging techniques of the human brain with behavioral outcomes, such as electroencephalography (EEG) and near-infrared spectroscopy (NIRS), helped to generate first valuable data in human exercise research. Increased muscle recruitment as revealed by electromyographic activity was accompanied by a higher brain cortical activity (measured by EEG) localized mainly in the primary motor cortices during unilateral isometric knee extensions (Abeln et al., 2013). This bilateral activation of motor cortex observed also in NIRS study (Derosière et al., 2014) during graded unimanual force production may be caused by the occurrence of interhemispheric inhibition (i.e., mirror activity of the passive limb during unilateral movement). Other brain regions, e.g., the mid/anterior insular cortex, were demonstrated by means of EEG to play an important role in the mediation of motor performance by exerting influence on motor regions (Hilty et al., 2011). To compensate for diminished force production capability, brain regions governing the motor behavior increase their couplings (Jiang et al., 2012). These studies indicate that coordination among brain regions is an important factor underpinning the behavioral manifestations of fatigue.

Another brain region involved in motor performance is the prefrontal cortex (PFC). The PFC involved in motivational and decision-making processes is an important candidate for acting as relay station in the central fatigue related network regulating central command. Development of fatigue at the highest force output during intermittent isometric upper limb contractions was attributed to reduced muscle oxygen availability rather than impaired prefrontal oxygenation (Bhambhani et al., 2014). Conversely, elevated metabolic demands during incremental cycling task (Racinais et al., 2014) affect both PFC and muscle oxygenation levels, and in turn, muscle recruitment responses. Indeed, the well-recognized PFC deoxygenation signature during the last stages of incremental exercise (quadradic trend, in Rooks et al., 2010) occurs concomitantly with an increased electromyographic activity (Racinais et al., 2014). Overall, these findings suggest that during whole body exercise but not during static exercise, there is inadequate delivery of oxygen to the brain. This could lead to altered PFC responses (i.e., executive function and cognitive control processes) to exercise.

In recent years, noninvasive brain stimulation techniques like transcranial direct current stimulation (tDCS) have also gained popularity owing to its potential to explore the neural processes underlying motor functions (Mauger, 2013). Recent reports used tDCS to produce beneficial effects on motor performance by attempting to reverse fatigue (Muthalib et al., 2013; Williams et al., 2013). Further development combining neuroimaging techniques with tDCS is needed in order to track reorganization changes across different levels of the nervous system during fatigue development.

## Post-exertion brain fatigue

Investigating the post-exercise recovery strategies could shed light on the contributions of the brain into the control of muscle force with fatigue development. Regarding the post-exercise recovery period, little or no data are available concerning central processes of performance (De Pauw et al., 2013). Assisting in brain recovery was recently highlighted (Minett and Duffield, 2014; Rattray et al., 2015). Main contributors on recovery processes in the brain following exercise are carbohydrate intake, temperature change, sleep quality, and modulation of brain activity with tDCS. Providing a window on the dynamics of human brain functioning under physiological conditions after exercise combining EEG with NIRS techniques is the next phase.

Recent evidences hold that the central nervous system ensures a significant role in the control of muscle force production in an extremely flexible manner both during exercise and in recovery. The potential utilities of complimentary and novel techniques (EEG-NIRS-tDCS) to understand cortical brain function during and after exercise in healthy humans should guide our future works.

### Conflict of interest statement

The author declares that the research was conducted in the absence of any commercial or financial relationships that could be construed as a potential conflict of interest.
